# Polymyositis anti-SRP antibodies and pregnancy about 2 cases

**DOI:** 10.11604/pamj.2016.24.192.9690

**Published:** 2016-07-01

**Authors:** Cissé Ousmane, Ba El Hadji Makhtar, Ba Fatoumata, Gams Daniel Massi, Daddah Sami Mouhamed Lémine, Diop-Sène Marieme Soda, Sow Adjaratou Dieynaba, Basse Anna, Seck Lala Bouna, Ndiaye Moustapha, Diop Amadou Gallo, Ndiaye Mouhamadou Mansour

**Affiliations:** 1 Department of Neurology, CHNU Fann, UCAD, Dakar, Sénégal; 2 Psychiatry Service CHNU Fann UCAD, Dakar, Sénégal; 3 Physiology Laboratory UGB de Saint Louis, Sénégal; 4 Neuro-Psychiatric Center in Nouakchott, Mauritanie

**Keywords:** Myopathy, anti-SRP antibodies, pregnancy, post-partum, corticosteroid

## Abstract

Anti-SRP myopathy represents 4 to 6% of all the inflammatory myopathies. It has been described since the 80s and its influence on pregnancy and vice versa has been highlighted recently. We report two cases of anti-SRP myopathy associated with pregnancy. In the first case, the initial manifestations of the disease started in post partum and the second case was an anti-SRP myopathy patient before pregnancy. In both cases we objectified outbreaks during post-partum. Pregnancy seems to promote outbreaks. The inactive myopathy seems to presents no serious maternal-fetal complications as well as the usual dose of corticosteroids. The treatment (corticosteroid) during pregnancy is indicated given the risk of worsening during the post-partum.

## Introduction

Anti-SRP myopathies are rare, they represent 4-6% of all inflammatory myopathies [1]. This is a condition characterized by symmetrically impairment of the proximal muscles with severe heterogeneous evolution, refractory to corticosteroid [2]. The association with pregnancy including post partum is rarely described in fact the first case was reported recently [2]. We report two cases of anti-SRP myopathy whose outbreaks occurred in the post-partum leading to the question of the role of pregnancy on their onset and vice versa. Indeed few studies have been done about this form.

## Patient and observation

### Observation 1

Ms. V.B. Caribbean 26 years (in 2012) with past history of Bypass in November 29^th^ 2009, uterine fibroid and normal delivery in March 2012. She has been hospitalized from the 14th to 21^st^ September for a chronic progressive muscle weakness of both lower limbs since mid-August. Clinical examination at admission revealed symmetrical paresis of both lower limbs with muscle strength quoted at 2/5 proximally and 3/5 distally with a positive Tabouret's sign, absence of knee jerk and ankle jerk reflexes with no idiomuscular response (deltoids and psoas muscles). There were no Babinski sign and no systemic signs. Laboratory tests showed increased CPK level (20,591 IU/L), positive C-reactive protein (15 mg/l) and erythrocyte sedimentation rate (13 at the first hour). The dosage of anti-parietal cell and anti-SRP antibodies were positives while the dosage of anti-JO1 and antinuclear antibodies were negatives except the insignificant presence of anti-SSA antibodies. Infectious serologies (HIV 1 and 2, HTLV 1 and 2, hepatitis A, hepatitis C, trichinosis, syphilis, Lyme, streptococci, Coxsackie virus, Echovirus) were negatives. We noted the presence of toxoplasmosis IgG (0.1 IU/ml) but no IgM. Epstein Barr virus serology revealed previous infection and we related the presence of anti-HBs antibodies (402 IU/l) to a previous immunization. The endocrine tests were normal (TSH, ACTH, estradiol, IGF1, prolactin, FSH, LH). The standard short synacthen test was negative. Cardiac ultrasound performed on the 17th September was normal. Magnetic Resonance Imaging (MRI) of the pelvis and thighs showed hypersignal of pelvis and thighs muscles indicate an inflammatory myopathy. The electromyogram showed pure myogenic pattern. We performed a surgical muscle biopsy at the left thigh which revealed muscular alterations related to inflammatory myopathy. The diagnosis of anti-SRP antibody myopathy was done.

Following the worsening of the muscle weakness, the patient was readmitted on the 05th September. Laboratory tests showed persistent high CPK level (18721 IU/L), elevated transaminases with AST (about 10 times) and ALT (167 IU/l), normal serum electrolytes, positive C - reactive protein (15 mg/l) and increased fibrinogen level (4.37). She received a treatment based on Methylprednisolone at initial dose of 500 mg and two others doses of 250 mg and Intravenous Immunoglobulin to 2g/kg/treatment. The treatment was well tolerated clinically and biologically (post-Intravenous Immunoglobulin CPK level was 7435 IU/l) but there were no regression of the muscle weakness. She has been discharged on the 12th October with corticosteroid and associated treatment.

### Observation 2

Ms. N.C. Senegalese 35 year old (in 2016) with past history of anti-SRP antibody myopathy diagnosed in 2012 and normal delivery. She was hospitalized from the 10th to 29th April 2015 for a muscle pain and weakness of the lower limbs started 5 months prior to hospitalization. Indeed seven months after the delivery, she has been re admitted for a muscle pain and weakness of the lower limbs leading to sleep deprivation. She has stopped the treatment during pregnancy without significant change. Clinical exam found muscle paresis of both lower limbs with muscular strength quoted at 1/5 proximally and 4/5 distally. The knee jerk, ankle jerk reflexes and idiomuscular response were absents bilaterally as well as the Babinski sign. Laboratory tests had showed high CPK level (17,651 IU/l), elevated transaminases with AST (about 10 times) and (ALT 140 IU/l), positive C-reactive protein (36mg/l), low creatinine level (75 mg/dL) and normal serum electrolyte.

Controlled laboratory tests done in January 2013 showed: very high CPK level (12,750 IU/l) with positive C-reactive protein 36 mg/l and erythrocyte sedimentation rate of 26 at first hour. The dosage of anti-SRP antibodies was positive while antinuclear antibodies and anti-JO1 antibodies were negative. Infectious serologies (HIV 1, 2, hepatitis B) were negative. The hormonal tests were normal (TSH, ACTH, prolactin, FSH, LH) as well as cardiac ultrasound. The electromyogram showed a pure myogenic pattern. She received Solumedrol with initial dose of 500 mg and two others doses of 250 mg. The treatment has been well tolerated clinically and biologically, without muscle paresis improvement. She has been discharged on corticosteroid and associated treatment.

## Discussion

The diagnosis of anti-SRP antibody myopathy was done based on the conditions chosen by Authier [3]: high CPK level; positive serum anti-SRP antibodies; a more proximal muscle weakness; no skin and joint manifestations; results of EMG, MRI and histology. The anti-SRP antibody myopathy only affects adults with a median age of 36 years (36 to 72 years). It is more common in women with a sex ratio of 0.2. [4] The association pregnancy and anti-SRP antibody myopathy has been rarely reported, the first case has been reported by Resseguier A.S. et al in 2013 [2]. This is seems to be due to the rarity of these conditions and high frequency of occurrence after 40 years [5]. Only 14% of patients are childbearing age at the diagnosis [6] but also by the severity of symptoms which can lead to advice against pregnancy. About 60 cases of pregnancies have been describe in patients with myopathies of different etiology [2].

Our first observation was anti-SRP antibodies myopathy whose signs appeared in the post partum. A similar case was reported by Ressenguer et al. [2]. The second case observation was a known anti-SRP antibodies myopathy patient who became pregnant. The outbreaks were observed during the postpartum for both patients, which raises the question of the effect of pregnancy on the occurrence of outbreaks. During pregnancy, there was no outbreaks despite the absence of treatment as reported by Resseguier et al. The recovery or the beginning of the active disease in postpartum one hand, the risk of maternal-fetal complication other hand raise the issue of the need for treatment even during pregnancy. Indeed, fetal prognosis is parallel to disease activity according to CQ Boch et al who published a more detailed series of pregnancy in 28 women with dermatomyositis and polymyositis and concluded that most myositis is active during pregnancy, the greater the risk of fetal complications is high [7]. Indeed, in case of remission, proceeds without complication generally unlike the active phase where it can be noted complicated with intrauterine growth retardation, prematurity [2].

This is consistent with our observation because none of our patients had no complications and maternal / fetal or. Treatment with corticosteroids usual dose in case of risk of maternal and fetal complications can be implemented [8]. Indeed the complications of this treatment may frequently [2] and the occurrence of postpartum pushed is a reality, our two patients had presented a pushed to the waning of childbirth with a worsening of symptoms in Case N° 2. the main complications of corticosteroids are not different from those observed in the general population with mainly diabetes, high blood pressure and infections [2]. Polymyositis does not increase labor complications in our patients because all have 2 vaginal delivery of a healthy child if that had required a cesarean is a woman 39 years old. [2]. A notable feature lab can probably reflect the active myopathic process was the determination of CPK returning higher. [9] This examination can be used to assess disease activity for therapeutic adjustment and reduce the risks driven in these patients. The anti-SRP antibodies appear to be correlated with CPK [10].

## Conclusion

Pregnancy seems to be a cause of genesis of outbreaks of polymyositis with anti-SRP antibodies. Given this fact and the possibility of maternal and fetal complications setting corticosteroids during pregnancy is an alternative for these patients.
